# “Air fresheners” as legal highs

**DOI:** 10.3325/cmj.2019.60.383

**Published:** 2019-08

**Authors:** Marina Bježančević, Katarina Dodig-Ćurković, Ružica Palić Kramarić

**Affiliations:** 1 University Health Center Osijek, Unit for Child and Adolescent Psychiatry, Osijek, Croatia; 2 School of Medicine, Josip Juraj Strossmayer University, Osijek, Croatia; 3 Faculty of Dental Medicine and Health in Osijek, Josip Juraj Strossmayer University, Osijek, Croatia; 4 Ministry of Health of the Republic of Croatia, Zagreb, Croatia *palickramaricruzica@gmail.com*

Similar to many other European countries, Croatia is witnessing the rise in the popularity of new psychotropic substances called legal highs. Currently in Europe there are over 450 monitored substances, with an emphasis on synthetic cannabinoids that mimic the effects of cannabis. They are legally sold in specialized stores, the so-called Smart shops, as air fresheners. These products are available under various marketing names (Galaxy, Atomix, Vertex, Bud Factory, Bonsai, Citrus...) and are labeled as not for human use. “Air fresheners” are herbal mixtures that are sprayed with synthetic cannabinoids and other new designer drugs.

Synthetic cannabinoids and their variants cannot be tested with routine drug tests. Their chemical content is diverse, unreliable, and unsafe, representing potential danger to the consumer. The term “designer drugs” indicates that chemically active ingredients of these products are constantly being modified to systematically avoid legal control. For example, when one substance is classified as prohibited, another one emerges, causing a similar effect, but its slightly different molecular structure makes it legal (1,2).

Young people, faced with constant indirect pressure from internet marketing, are progressively reaching for these substances. An increase in “air fresheners” consumption is associated with desirable negative results of routine drug urine testing, curiosity to try new drugs, affordable price, an easy access, and legal status, which all creates an illusion that these substances are safe for consumption (2,3).

The Office for Combating Drug Abuse of the Croatian Government indicates that newer generations of psychoactive substances are becoming increasingly toxic: in the period from 2014 to 2016, 43 poisonings and 1 death were reported in Croatia, most of them associated with a synthetic cannabinoid known as Galaxy. In 2017, the Center for Forensic Testing, Research and Expertise “Ivan Vučetić” identified 18 new psychoactive substances in Croatia.

## PHARMACOLOGY OF SYNTHETIC CANNABINOIDS

Synthetic cannabinoids were developed when the endocannabinoid system was studied under laboratory conditions. They are functionally, but not structurally, similar to the plant 9-tetrahydrocannabiol (delta-9-HTC), the active substance of cannabis (4). They bind to type 1 (CB1) receptors, the most common receptors in the brain, whose activation leads to psychoactive effects, and to type 2 (CB2) cannabinoid receptors, which are peripherally distributed and modulate the immune function. Unlike tetrahydrocannabiol, which acts as a partial CB1 receptor agonist, they have high potency for the same receptor and act as full agonists, which could explain their prolonged psychoactive effects (5).

## CLINICAL FEATURES

The synthetic cannabinoids intoxication has a very diverse clinical presentation, with a possibility of fatal outcome as a result of an overdose. Intoxication effects are similar to those of cannabis: relaxation, altered consciousness, disinhibition, or euphoria. The most common physical symptoms are rapid heartbeat, hypertension, difficulty breathing, fever and redness, nausea, muscle spasm, anger outbursts, overreacting, appetite disorder, and sleep disturbance, while psychological symptoms include depersonalization, derealization, panic attacks, severe anxiety, hallucinatory experiences, and paranoid interpretations of reality (6). Acute effects usually last from 30 to 120 minutes, but symptoms can persist until the next day or sometimes for weeks. Synthetic cannabinoids can trigger the development of a psychotic decompensation, especially in individuals with a family history of psychiatric disorder (7).

## OUR EXPERIENCE

At our Child and Adolescent Psychiatry Unit, we observed an increase in hospitalizations following intoxications with “air fresheners:” from one case in 2017 to as many as 13 cases in 2018. Considering very diverse clinical presentation and symptoms severity, we made a media appeal in leading Croatian newspapers to parents, children, and all relevant structures responsible for preventing the sale of these substances. The aim of our effort was to familiarize the parents with the term “air fresheners” and emphasize the need for an increased supervision of children's behavior.

## RECOMMENDATIONS

There is a need for the prevention of addiction diseases, education, as well as regulation and supervision of the stores that sell “air fresheners,” together with stricter legislation and penalties. The primary focus should be on early prevention, especially during early school years, with an aim of informing young people about the harmful effects of such substances and about the ways that they encourage addictive behavior. In today’s society, the increasing support for the legalization of psychoactive substances may convey the message that these substances have healing rather than negative health effects. In addition to the preventive actions, it is necessary to improve the education of parents/caregivers, as well as of all professionals working with young people. This is especially important in the school system, since peers often act as mediators and reinforce addictive behavior. It is also important to have strong legislation that will quickly remove the new designer drugs from the market, prohibit them, and clearly define the legal consequences for both the consumer and the seller.

We should continue to raise the awareness of the dangers of new narcotics and their adverse effects on children's health and, at the same time, promote healthy ways of relaxation among young people (playgrounds rather than video games, recreational camps, etc). Efforts should be made to increase family bonding, since the loss of support and insecure attachment could render young people vulnerable, making them turn to the world of designer drugs. Similar to the efforts made in Scandinavian countries, volunteer and charitable work should be encouraged among young people (child care institutions, elderly homes etc) with the aim of preserving the values such as empathy, helping, mutual understanding, cooperation, and support.
